# Laparoscopic Hartmann Procedure—A Surgery That Still Saves Lives

**DOI:** 10.3390/life13040914

**Published:** 2023-03-31

**Authors:** Costel Bradea, Eugen Tarcoveanu, Valentina Munteanu, Cristian Dumitru Lupascu, Florina Delia Andriesi-Rusu, Delia Gabriela Ciobanu, Alin Mihai Vasilescu

**Affiliations:** 1 First Surgical Clinic, “St. Spiridon” Hospital Iasi, Independentei Str., No 1, 700111 Iasi, Romania; 2 Faculty of Medicine, “Grigore T. Popa” University of Medicine and Pharmacy Iasi, 700115 Iasi, Romania

**Keywords:** laparoscopy, colorectal neoplasia, Hartmann procedure

## Abstract

Background: A Hartmann operation, which is the intervention by which the lower part of the sigmoid and the upper part of the rectum are resected with the closing of the rectal stump and end colostomy, has as its indications: advanced or complicated rectosigmoid neoplasm, moderate biological condition of the patient, peritoneal sepsis, intestinal occlusion and fragile colonic wall, especially in the context of inflammatory changes. The Hartmann procedure can save lives even at the cost of a stoma reversal failure. Methods: The cases operated with the Hartmann procedure by an open approach or laparoscopic approach in our clinic, between 1 January 2016 and 31 December 2020, were admitted in this study and their medical records were reviewed, also making a comparison between the two types of approach. Univariate statistical comparisons but also a multivariate analysis was performed. Results: We performed 985 operations for intestinal and colonic occlusion (7.15% of the total operations in the clinic), 531 (54%) were non-tumor occlusions and 454 (46%) were occlusive tumors (88 Hartmann operations). Of these, 7.3% were laparoscopically performed (7 laparoscopic Hartmann operations and 23 diagnostic laparoscopies). A total of 11 cases (18%) also had colonic perforation. We compared laparoscopic Hartmann with open Hartmann and observed the benefits of laparoscopy for postoperative morbidity and mortality. The presence of pulmonary and cardiac morbidities is associated with the occurrence of general postoperative morbidities, while peritonitis is statistically significantly associated with the occurrence of local complications that are absent after the laparoscopic approach. Conclusions: The Hartmann procedure is still nowadays an operation widely used in emergency situations. Laparoscopy may become standard for the Hartmann procedure and reversal of the Hartmann procedure, but the percentage of laparoscopy remains low due to advanced or complicated colorectal cancer, poor general condition both at the first and second intervention, and the difficulties of reversal of the Hartmann procedure.

## 1. Introduction

The Hartmann operation represents the intervention by which the lower part of the sigmoid and the upper part of the rectum are resected with the closing of the rectal stump and bringing the proximal part into colostomy. In the 18th century, the anatomist Giovanni Morgagni, from the school of Padua, proposed a rectal resection for cancer for the first time. In 1921, the French surgeon Henri Albert Hartmann described the operation of recto-sigmoid resection with provisional left iliac terminal colostomy for cancer [1]. In 1950, Boyden proposed the same procedure for diverticulitis [2]. In 1986, RJ “Bill” Heald published the new technique of total mesorectal excision, with positive results in 115 cases with curative resections (80% disease-free interval at 5 years and 3.7% relapse at 5 years and, until then, the relapse rate was 80% [3]). The indications for the Hartmann operation, a true lifesaving surgical operation, are moderately altered biological status of the patient, peritoneal sepsis, intestinal occlusion and fragile intestinal wall, usually due to rectosigmoid neoplasia or diverticulitis. Morbidity is increased and is consistent with associated cardiorespiratory disease and sepsis [4,5,6].

The Hartmann operation remains a popular surgical intervention in emergencies. It is comparable even more favorable than other surgical operations (colostomy, resection, anastomosis). The major advantages of the Hartmann procedure are the immediate resection of the affected colorectal segment and avoiding the risk of anastomotic fistula. The disadvantage of the Hartmann operation is the high percentage of failure of colostomy reversal following the Hartmann Procedure (Hartmann II) of 40%, as well as the high percentage of morbidity (34%) after Hartmann II [7]. Laparoscopic surgery improves the outcomes, mortality, early overall morbidity, major and minor complications, surgical complications and postoperative hospital stay.

Today, developments in anesthesia, antibiotic therapy, intensive care and surgery, as well as colonic lavage on the operating table, have facilitated resection and anastomosis in a single operation, with reduced morbidity and mortality [8,9,10,11]. However, septic shock, fecal peritonitis, immunocompromised state or ASA IV are contraindications for anastomosis. Under these suboptimal conditions, the Hartmann procedure can save lives and is still considered safe and valid [12,13,14].

## 2. Methods

The cases operated with the Hartmann procedure by open approach or laparoscopic approach in our clinic, between 1 January 2016 and 31 December 2020, were admitted in this study and their medical records were reviewed, also making a comparison between the 2 types of approach. Univariate statistical comparisons but also a multivariate analysis was performed with the statistical analysis program SPSS. The statistically significant p was considered at a value <0.05. We compared the 58 cases with classic Hartmann with the 30 cases of Hartmann with laparoscopy (7 Hartmann laparoscopic and 23 Hartmann with laparoscopy and conversion). The laparoscopy gives a better condition for diagnosis (for tumors, hepatic metastases or peritoneal metastases) and treatment (dissection and way of resection).

In the Hartmann surgery technique used in our clinic, the patient is placed in a modified lithotomy position, with the lower limbs in slight flexion. This position allows the transanal introduction of a circular stapler (in case primary anastomosis would be possible), offers the possibility of intraoperative colonoscopy and the positioning of the surgeon between the lower limbs of the patient during the descent of the left colic angle in the Hartmann I or II operation.

A transanal rigid or flexible rectoscope may be used to confirm that the resection margins are adequate, non-inflamed, and that there are no diverticula or tumors, or that the anastomosis is correct. The surgeon will sit to the right of the patient, with the first aid in front of him and the second aid between the lower limbs of the patient. For laparoscopy, two monitors must be used, which are placed on either side of the patient’s legs.

The advanced laparoscopy kit containing a 30-degree lateral view laparoscope, disposable or metal trocars, Hasson trocar for open laparoscopy, longer and stronger instruments, visceral retractors, atraumatic Babcock forceps, hook, electric scissors, bipolar coagulation forceps (for hemostasis) and ultrasound (for dissection), laparoscopic needle holders, clip applier, laparoscopic stapler (60 mm or 45 mm articulated endoGIA stapler, the latter easier to handle in the narrow pelvis), VILOCK sutures, endo-roeder loop, lavage cannula, endo-bag, abdominal wall protection bag and drain tube. For better orientation, it would be ideal if the operator, the camera, the work field and the monitor are placed on the same line.

Standard oncological principles are followed: early proximal vascular ligation of the tumor segment to be resected, establishing the margins of the distal and proximal section, and appropriate lymphadenectomy with the mesorectal excision.

Pneumoperitoneum in the laparoscopic approach can be performed with the Veress needle and the optical umbilical trocar is “blind” inserted. Open laparoscopy is preferred through the Hasson trocar (which is inserted through a mini-laparotomy, at the umbilicus or away from the postoperative scars and is attached to the abdominal wall through the two sleeves around it).

Laparoscopic staging gives us data on tumor extension and liver or peritoneal metastases (in principle, we have abdominal CT (even in emergency at the UPU) which shows tumor penetration and adenopathy). If the tumor is hemorrhagic or perforated, it is indicated to perform a colorectal resection, even if the stage is locally advanced or metastatic.

All seven spaces of the peritoneal cavity are controlled with the laparoscope, assisted, if necessary, by the removal and unwinding of the viscera with “goose paw” forceps and spacers, with dissection with the hook, “cold” or electric scissors, with bipolar or ultrasound forceps.

**Figure 1:**
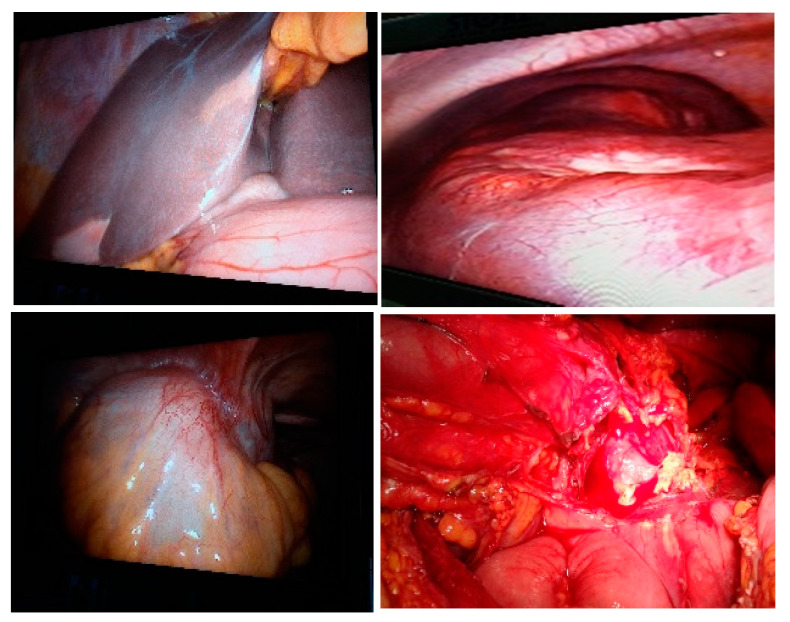
Diagnostic laparoscopy and conversion—the presence of liver metastases (**top left** and **right**), parietal invasion (**bottom left**) or perforated tumor (**bottom right**) is observed.

When the recto-sigmoid tumor is occlusive, in stage IV, only the biopsy of the peritoneal or liver metastasis is performed, as well as the left lateral iliac colostomy under video control (the Asian model of tumor and metastatic resection is also suitable in patients with acceptable general condition). If the recto-sigmoid tumor is locally advanced, resectable together with the invaded viscera, a multivisceral “en bloc” classic resection is performed by conversion (extended Hartmann operation) (Figure 1).

If we judge that a classic or laparoscopic Hartmann resection is feasible, we will start by sectioning the peritoneum from the para-colorectal troughs with the hook or scissors. We take care of the ureters (we can put them on a loop) and the hypogastric nerve network, especially during retroperitoneal dissection for lymphadenectomy in Heald’s “holy” plane (between the colorectal fascia and the pelvic fascia).

The surgeon can stay to the right of the patient and use their right hand for dissection and have their left hand on the telescope (the assistant will pull the colorectum with the Babcock forceps) in the case of laparoscopy, or the surgeon will stay to the left of the patient using their hands for traction and dissection, detaching the rectum against the pelvic wall with the Ligasure forceps, microwaves, scissors or hook. The resection is performed “en bloc”. The meso-colon and meso-rectum are resected up to 5 cm below the tumor, achieving R0.

**Figure 2:**
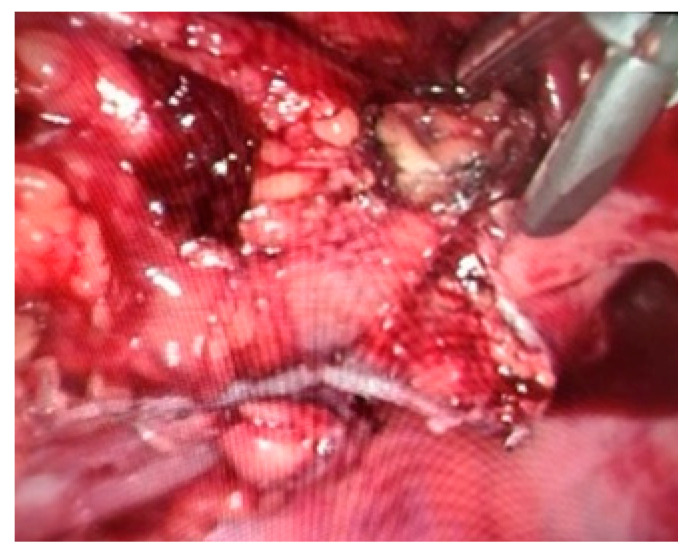
Intraoperative view—Mesorect excision with Ligasure device.

Colic and rectal vessels can be clipped for more safety. In general, the 10 mm Ligasure forceps ensure good hemostasis without clips (Figure 2).

**Figure 3:**
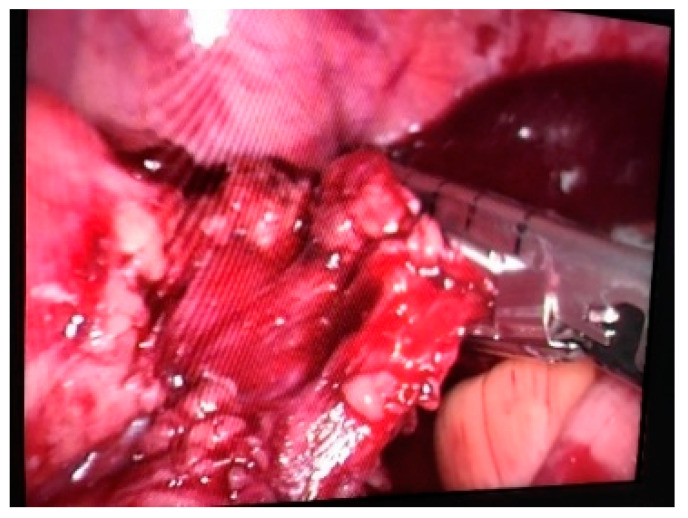
Intraoperative view—Rectal section with Endo GIA stapler.

After the dissection, there is time to section the digestive tube with a blue or green stapler, 60 mm, articulated, 5 cm below the tumor (intraoperative endoscopy can be useful, if we do not notice the tumor) (Figure 3).

**Figure 4:**
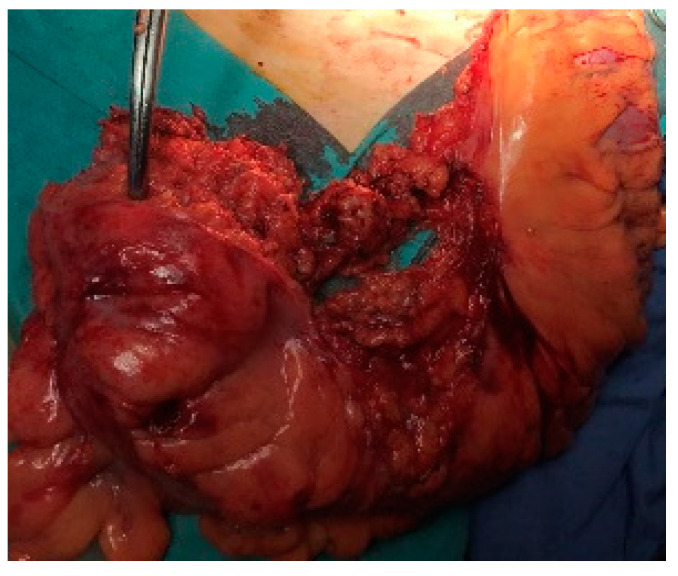
The colorectum to be resected is exteriorized through the trocar hole in the left iliac fossa.

The colorectum to be resected is exteriorized through the trocar hole in the left iliac fossa, placed on the colostomy site, by traction with the Babcock forceps, with a protective bag for the abdominal wall (Figure 4).

**Figure 5:**
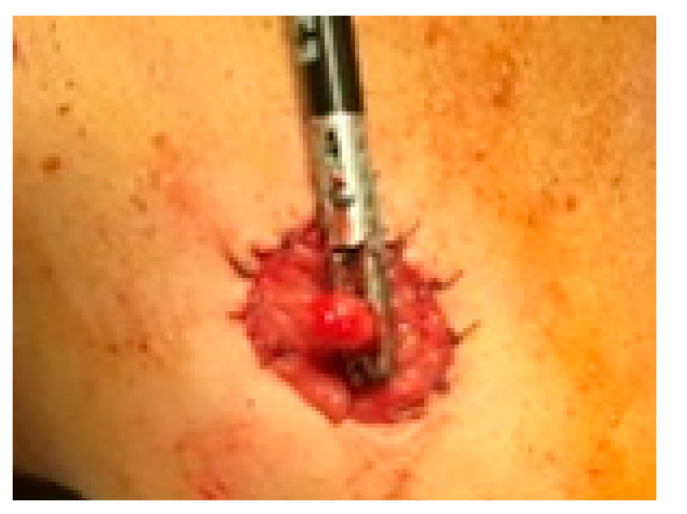
Colostomy—resection of a polyp located on the stomatized colic loop is performed.

The colon is sectioned 10 cm above the tumor and fixed in a provisional left iliac terminal colostomy (Figure 5).

The peritoneal cavity is rechecked classically or laparoscopically and the breach behind the colostomy is closed with a suture.

**Figure 6:**
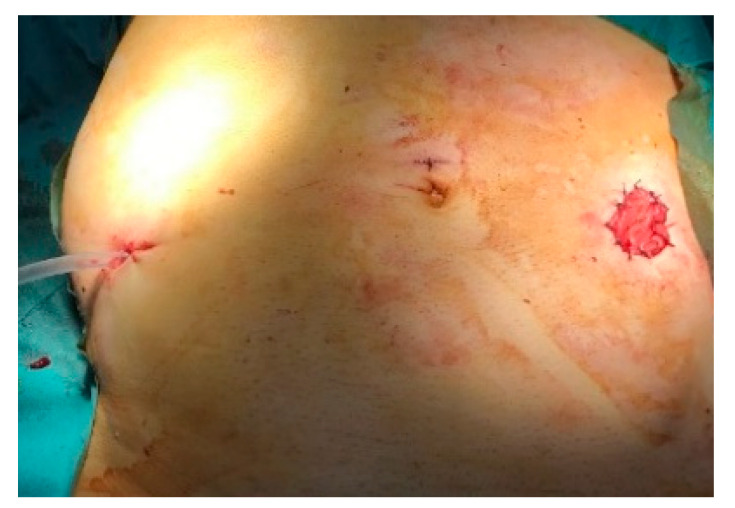
Laparoscopic Hartmann procedure—a drain tube is exteriorized through the trocar hole in the right iliac fossa.

A drain tube is installed in the space of Douglas, exteriorized through the trocar hole in the right iliac fossa (Figure 6).

**Figure 7:**
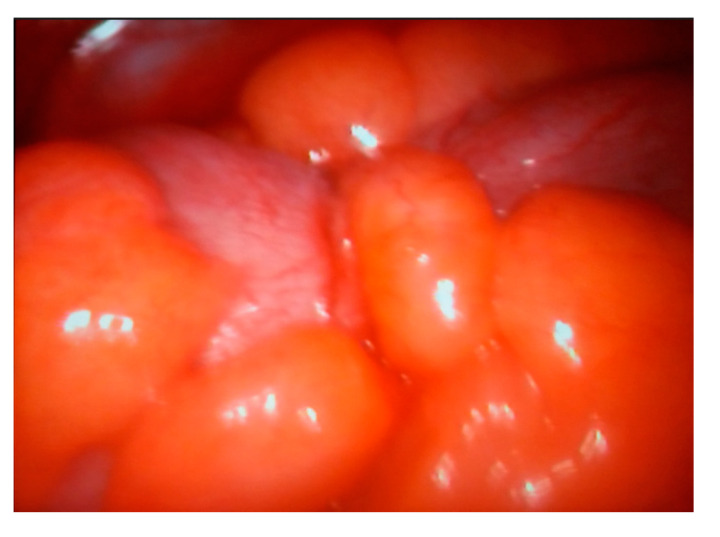
Diagnostic laparoscopy—advanced tumor.

**Figure 8:**
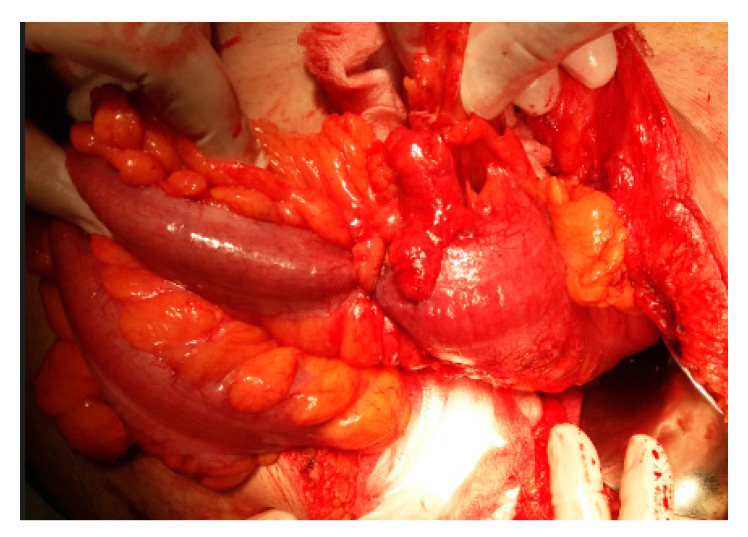
Intraoperative view—conversion for advanced tumor.

If it is estimated that there are no conditions for laparoscopy due to the local conditions of the tumor, the conversion to open surgery is made (Figure 7 and Figure 8).

A few months after the Hartmann operation (6 months after colorectal cancer), the rectum can be put back into the circuit by mechanical or manual, classical or laparoscopic colorectal anastomosis.

**Figure 9:**
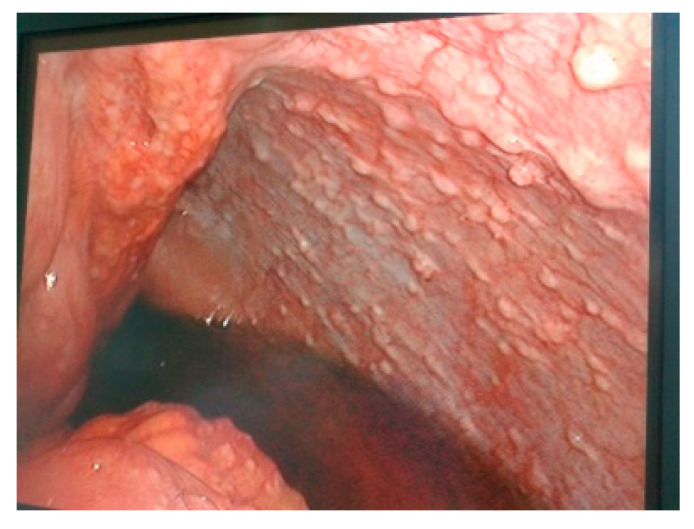
Intraoperative view—peritoneal carcinomatosis.

It starts with diagnostic laparoscopy to assess the feasibility (for hepatic, peritoneal metastases or postoperative adhesions) (Figure 9) through the Hasson trocar and accessory trocars, preferably on the same scars from the laparoscopic or classical Hartmann operation.

**Figure 10:**
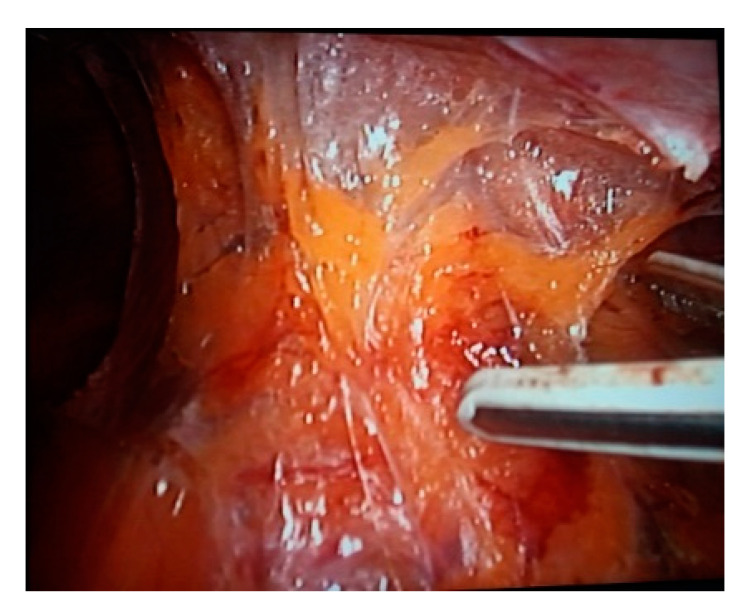
Intraoperative view—adhesiolysis with Ligasure device.

Adhesiolysis is performed with the hook, “cold” or electric scissors, Ligasure or ultrasound (Figure 10).

**Figure 11:**
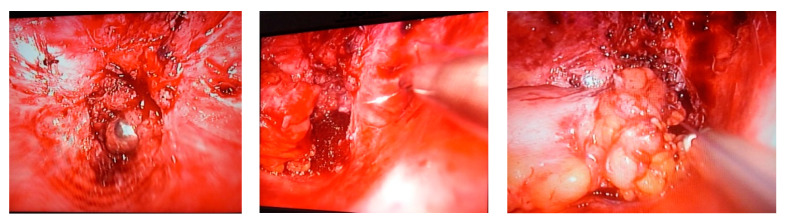
Laparoscopic colorectal anastomosis in the Hartmann II operation—the rectal stump is highlighted, suture and final aspect (from left to right).

The rectal stump is highlighted; the colostomy loop is cut from the abdominal wall, and laparoscopic intracorporeal colorectal anastomosis is performed with separate stiches or intraabdominal suture or with extracorporeal Roeder’s knot (Figure 11).

The laparoscopic ability, in the two operations, Hartmann I and II, in order to respect the oncological principles depends on the size of the tumor and its location, and on anatomical factors such as a narrow pelvis, obesity, large uterus, as well as the effects of preoperative radiotherapy. The inability must lead at conversion to prevent incidents, complications or incomplete resection.

### 2.1. Technical Variations

The piece can be extracted transanally or through a Pfannenstiel incision. The lower mesenteric vessels can be approached and sealed, starting with the Treitz ligament, then dissection on the cranio-caudal Told and Gerota fascia, especially if we know for sure that it is a malignant lesion. Pre- and per-laparoscopic colonoscopy establishes the topography of the colorectal tumor and allows it to be tattooed to be more easily visible. Pelvic exenteration can be performed “en bloc” if the colorectal tumor invades the surrounding organs (bladder, prostate or uterus/vagina).

Robotic surgery is feasible and safe for colorectal resection in selected patients. This surgery overcomes the disadvantages of laparoscopic surgery: dependence on the cameraman, 2D vision, instruments with fixed and immobile tips, impossible to use in dihedral angles, surgeon tremors and lack of ergonomics.

Intraoperative Indocyanine Green fluoroscopy can be used to detect sentinel adenopathy.

The “hand assisted” method is a bridge between classical and laparoscopic; it reduces the learning curve and makes it easier. The possibility of using the surgeon’s sense of touch is kept, and manual dissection and operative time are reduced.

Sigmoidectomy can be performed transanally for fragile patients: a bursa is made, transanally, under the tumor, tight (Roeder’s node); the rectum is sectioned on the posterior wall from the lumen towards the retrorectal space until it reaches the peritoneal cavity; the mesocolic or mesorectum is resected as well as the tumor with oncological safety limits; transanal laparoscopy is also performed for the inspection of the entire peritoneal cavity; the colon above the tumor is sectioned with the Endo-GIA linear stapler passed transanally and the resection piece is extracted also transanally. Then, the colostomy is made and the rectal section is closed.

In patients who also undergo laparoscopy, the above method can be video-assisted with a telescope and forceps passed through transabdominal trocars.

To prevent para-stomal hernia, we can add a prevention procedure with videoscopically applied dual mesh (Sugarbaker) or implant the mesh around the colostomy hole “buried” in the abdominal muscle sheath.

### 2.2. Incidents and Intraoperative Accidents

Lesions of the vessels and ureteral lesions may occur.

Laparoscopic colectomies have a conversion rate of 8 to 40%, with a high possibility of parietal contamination in malignant lesions.

The conversion is imposed by technical difficulties, uncertain anatomy, hemorrhage, intestinal injuries or other viscera.

### 2.3. Postoperative Care and Complications

They are common to classical and laparoscopic surgery or only to laparoscopy (parietal and visceral lesions caused by the trocar, postoperative hernias at the level of the trocar holes, hypercarbia, bradyarrhythmia, pneumoepiploon) and specific (anastomotic fistulas, stenosis, ureteral lesions).

There is talk of seeding the trocar sites with tumor cells due to the gas under pressure from the laparoscopy.

Laparoscopic colorectal surgery is much more difficult; it requires expensive equipment and instruments, special experience and special training.

Postoperative evolution is marked by less discomfort, faster resumption of transit and early feeding. The benefits of laparoscopy consist in the reduction of postoperative pain, cosmetic appearance, early mobilization and quick resumption of activity.

The prognosis is dependent on the condition for which the intervention was performed.

## 3. Results

**Figure 12:**
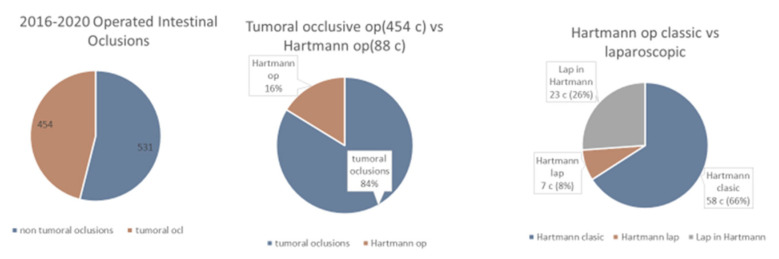
Operations for intestinal and colonic occlusion.

At The First Surgery Clinic, between 2016 and 2020, out of 985 operations for intestinal and colonic occlusion (7.15% of the total operations in the clinic), 531 (54%) were non-tumor occlusions and 454 (46%) were occlusive tumors (88 Hartmann operations). Of these, 7.3% were laparoscopically performed (7 laparoscopic Hartmann operations and 23 were diagnostic laparoscopies) (Figure 12).

In 2016, there were 238 operations for intestinal occlusion (115 (48.3%) tumor occlusions, of which 24 were Hartmann operations, 123 (51.7%) non-tumor occlusions, of which 16 (6.7%) were laparoscopic (1 case Hartmann operation and 1 case diagnostic laparoscopy).

In 2017, 172 occlusions were operated (84 tumor occlusions (48.8%) of which 18 were classical Hartmann operations, 88 non-tumoral occlusions (51.2%) of which 6 were laparoscopic (3.5%), and of which one case was with diagnostic laparoscopy and one case with Hartmann surgery).

In 2018, 178 occlusions were operated (72 (40.44%) tumor occlusions, of which 11 Hartmann, 106 (59.56%) non-tumor occlusions, of which 10 were laparoscopies (5.81%) (one case of laparoscopy followed by Hartmann operation).

In 2019, 139 patients with intestinal occlusion were operated on (109 (43.09%) tumoral occlusions, of which 17 were Hartmann and 130 (56.10%) were non-tumoral occlusions, of which there were 33 laparoscopies (13.80%), and of which 4 diagnostic laparoscopies were before the Hartmann operation and 4 were laparoscopic Hartmann). It was the most prolific year.

On the other hand, in 2020, due to the COVID-19 pandemic, the addressability was low: 158 occlusions, 74 (46.83%) tumoral occlusions, of which 12 were Hartmann operations and 84 (53.17%) were non-tumoral occlusions), of which 7 (4.43%) were laparoscopies (of which 4 laparoscopies were before the Hartmann operation).

**Figure 13:**
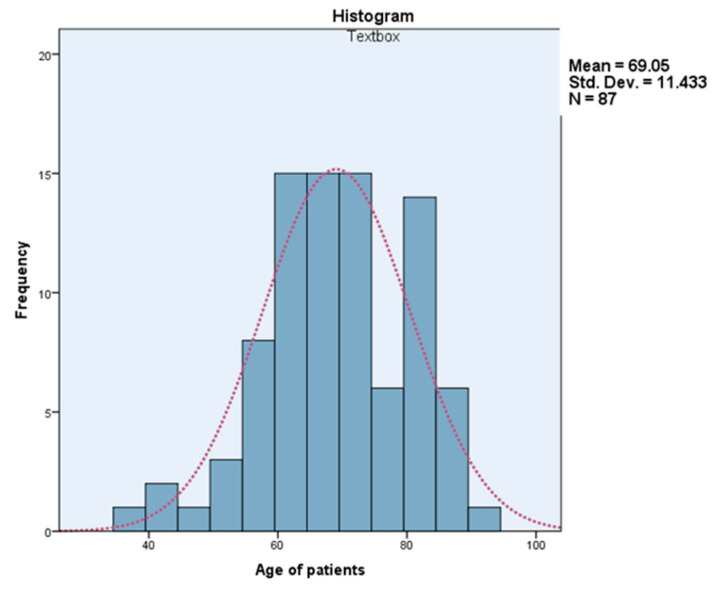
The mean age for laparoscopic Hartamnn procedure.

The mean age of patients operated on with intestinal occlusion in the years 2016–2020 in our clinic was 70 years old for the Hartmann open approach for tumor occlusion (range 37–91 years), 80 years old for classically operated non-tumoral occlusion and 69 years for those laparoscopically operated for both tumoral and non-tumoral occlusion. The mean age for laparoscopic Hartmann procedure was 69 ± 11.43 years (range 37–91 years) (Figure 13).

The male/female sex ratio for classically operated tumor occlusion was 4/6 (for Hartmann 6/4); 5/5 for classic non-tumor occlusions; 65/35 for laparoscopies for tumor occlusion; and 5/5 for laparoscopies for non-tumor occlusion. The study of operations per year (percentage), by sex, showed a relatively equal distribution.

The patients with Hartmann surgery were from the urban environment in 57.47% of cases (50 cases).

There were 72 laparoscopies for occlusion, of which 60 (83.33%) discovered occlusive tumors (23 diagnostic laparoscopies before Hartmann and 7 laparoscopic Hartmann) and 12 (16.66%) for non-tumor occlusions.

The greatest number of laparoscopies were performed in 2019 and the fewest in 2020 due to the COVID-19 pandemic which brought low addressability.

In 2016, out of 16 laparoscopies, 15 (93.75%) were for tumor occlusions (1 diagnostic laparoscopy before classical Hartmann and 1 case of laparoscopic Hartmann) and 1 (6.25%) was a non-tumoral case. In 2017, out of six laparoscopies, five (83.33%) were for tumor occlusion (one case of diagnostic laparoscopy before classical Hartmann) and one case (16.66%) for non-tumor occlusion. In 2018, out of ten laparoscopies, eight (75%) were for tumor occlusions (one case of diagnostic laparoscopy before classic Hartmann surgery) and two cases (25%) for non-tumor occlusion. In 2019, out of 33 laparoscopies for occlusion (5 diagnostic laparoscopies before classic Hartmann and 5 Hartmann celio), 26 (78.78%) were for tumors and 7 (21.12%) non-tumoral. In 2020, there were only 7 laparoscopies because laparoscopy was prohibited during the initial period of the COVID-19 pandemic. There were six (85.71%) for tumor occlusion (four diagnostic laparoscopies before the classical Hartmann operation) and one case (14, 19%) non-tumoral. Most patients had one or more comorbidities: cardiopathies 60%, 57 (65.51%); hepatopathies 40%, 33 (38%); 30% of those operated for intestinal occlusion had anemia; pulmonary diseases were preoperative in 10%; and 80% of those who underwent classical surgery had other comorbidities.

The statistical processing proves that the presence of pulmonary and cardiac morbidities is statistically significantly associated with the occurrence of general complications (Table 1). The presence of cardiac comorbidities increased the rate of general postoperative complications; the chi-square test showed a statistically significant association. The rate of general postoperative complications was seven times higher in the group of patients with cardiac comorbidities. At the same time, there is also a statistical association between the presence of cardiac comorbidities and the rate of local postoperative complications (chi-square *χ*^2^ test, *p* = 0.031). There is also a statistically significant association between the presence of pulmonary comorbidities and the occurrence of general complications. The incidence of general complications was 2.5 times higher in the group of patients with pulmonary comorbidities.

In statistical processing, peritonitis is statistically significantly associated with local complications (Table 2).

The statistical analysis shows that more than 80% of the patients who presented with peritonitis developed local postoperative complications, highlighting an important statistical association between the presence of peritonitis and the increase in the incidence of local postoperative complications.

There is no statistically significant association between the presence of peritonitis at admission and the occurrence of general postoperative complications (chi-square *χ*^2^ test, *p* = 0.138).

There is no statistically significant association between the presence of occlusion and the occurrence of local (chi-square *χ*^2^ test, *p* = 0.646) or general postoperative complications (chi-square *χ*^2^ test, *p* = 0.803); although, they were more numerous in the occlusion group.

**Figure 14:**
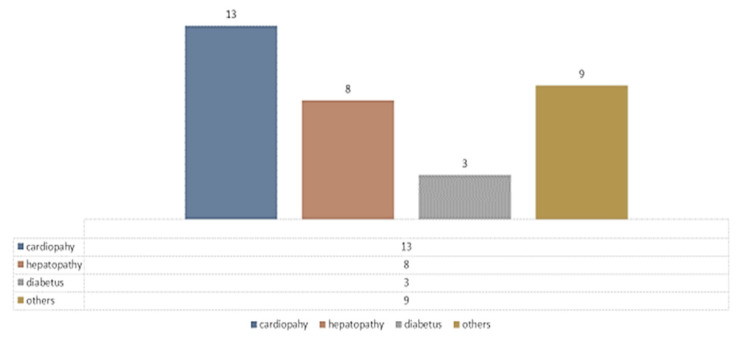
Hartmann comorbidities distribution.

ASA was, for in the majority of patients, in risk class III (for Hartmann, ASA III risk = 49c (56.30%), ASA II = 32c (36.78%)). The distribution of preoperative comorbidities in Hartmann patients can be seen in Figure 14, where heart disease and hypertension predominate.

**Figure 15:**
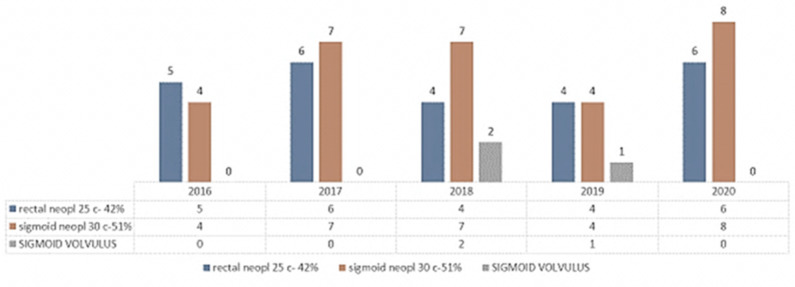
Sigmoid/rectal cancer parity.

The parity of sigmoid cancer/rectal cancer was according to Figure 15, where it can be seen that sigmoid neoplasms tend to exceed rectal ones in patients who came to the emergency room and underwent Hartmann surgery.

**Figure 16:**
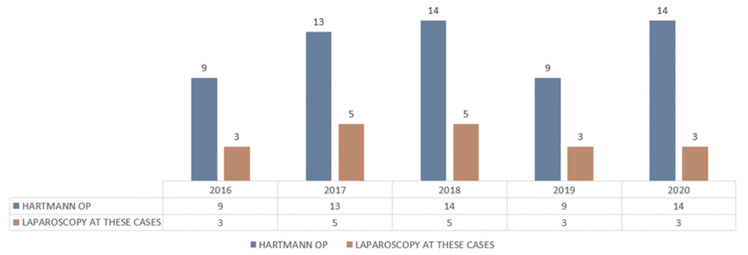
Open vs. laparoscopic Hartmann procedure parity.

The parity of the open Hartmann procedure versus laparoscopic Hartmann procedure is shown in Figure 16.

**Figure 17:**
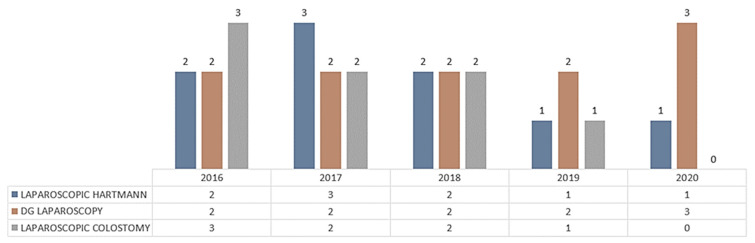
Laparoscopy in Hartmann colorectal resection vs diagnostic laparoscopy vs video-assisted colostomy.

Routine laparoscopic staging tends to be performed to avoid unnecessary laparotomy in oncologically advanced stages by performing diagnostic laparoscopy, video-assisted colostomy, or performing laparoscopic Hartmann surgery (less in 2020 due to COVID-19 when laparoscopy was banned) (Figure 17).

Not all patients could be put back in the digestive circuit due to age and comorbidities, adhesions or patients who do not present for follow-up. Among the tumors operated on by Hartmann procedure, 14% were G1, 36% G2 and 50% G3. It is commendable that 80% of Hartmann procedures were accompanied by lymph node excision with more than 15 nodules, which represents a high oncological quality.

Looking at the TNM classification, Colo Rectal Carcinoma (CRC) operated with Hartmann procedure was mostly in stages T3 (43%), T4 (36%) and N0 (31%), N2 (33%), M0 (93%). We expected that in the emergency situation, the tumor and lymph node invasion are advanced, and the Hartmann operation protects from relapse on the anastomosis.

**Figure 18:**
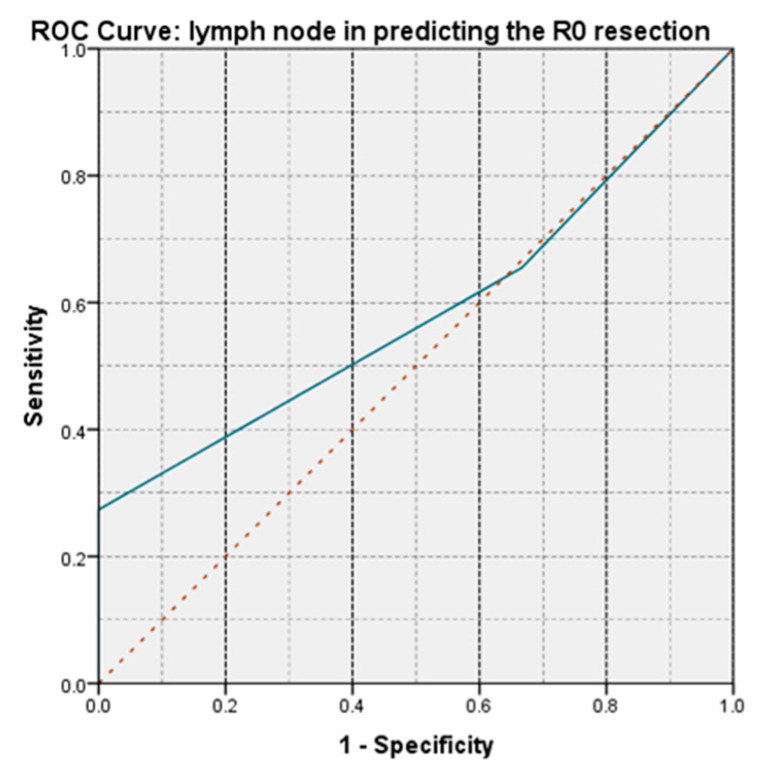
The aspect of the ROC curve for lymph node invasion shows a low power of prediction for obtaining an R0 resection.

For all patients with N0, an R0 resection was obtained. The aspect of the ROC curve and the value of AUC = 0.585 demonstrate that lymph node invasion has a low-moderate power of prediction on obtaining an R0 resection (Figure 18).

Those operated with the Hartmann procedure had 28% local complications, 43% general complications and 29% had no morbidities. Half of those classically operated for intestinal occlusion had an evolution without morbidities, and of those, laparoscopically, 80% had an evolution without complications. Postoperative mortality in those with intestinal occlusion was 10%. Among those who underwent Hartmann surgery, the mortality rate was 21%.

The occlusive tumors were generally in the T3 stage (60% of those operated classically as well as laparoscopically). The degree of differentiation G2 (moderately differentiated) was found in 70% of those operated classically and 90% of those operated laparoscopically.

**Figure 19:**
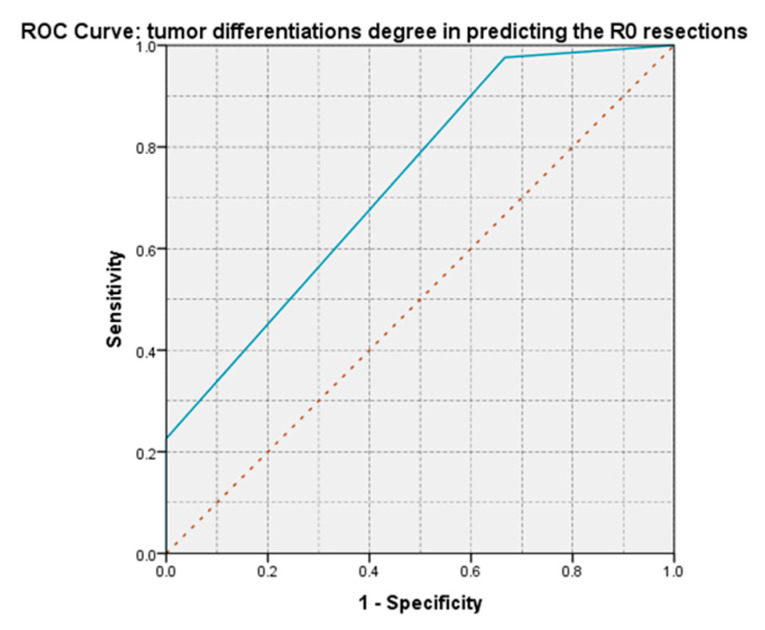
The aspect of the ROC curve for the degree of tumor differentiation shows a moderate predictive power on obtaining an R0 resection.

For all patients with well-differentiated tumors, an R0 resection was obtained, while for those with poorly differentiated tumors, this was possible in a proportion of 6.66%. The appearance of the ROC curve and the AUC = 0.730 value demonstrate the degree of tumor differentiation; it has a moderate predictive power on obtaining an R0 resection and can be used as a predictive marker (Figure 19).

The results of the statistical analysis did not highlight any statistically significant difference between the number of nodes excised and the surgical approach chosen. The number of excised nodes varied between 12 and 56, with an average number of 19 for open surgery and 22 for laparoscopic surgery (Table 3).

Regarding the number of days of hospital stay, there is an important statistical difference, with a *p* = 0.015 between the laparoscopic approach and the open approach; the number of days of hospital stay was significantly lower in the case of the laparoscopic approach, with an average of 5 days and a maximum of 8 days. The average number of days of hospitalization was 12 days for open surgery (Table 4).

## 4. Discussions

Compared to other authors, our study is part of the general trend, but CRC predominates, 93% vs. 59% (Leong, Singapore) vs. 32% (Hallam, UK), with comparable rates in hospital stay, and postoperative mortality (Table 5) [12,15,16,17,18].

Our study and other studies show that mortality was affected by preexisting respiratory or cardiac diseases, age and ASA grade, and, likewise, morbidity increases significantly in the presence of cardiac comorbidities and age. Diabetes, renal failure, cardiovascular disease, immunodeficiency and malnutrition can affect the anastomosis [14]. Most patients with colorectal cancers are elderly and have at least one of these comorbidities with a high ASA risk of III, so a Hartmann operation is often the safest option [17,18,19,20].

The Asian population has a high percentage of colorectal cancer as an indication for Hartmann Surgery compared to Western countries, but still the mortality results are not worse; however, the morbidity is higher due to the recurrence and the occlusive or perforating complication of the cancer. Most patients were elderly and had comorbidities [12,19].

The average duration of hospitalization of 12 days, although all cases were operated on in an emergency and most patients were in poor general condition, is similar in most other studies [12,16,17,21,22].

The mortality of 19% in our study is similar to other studies, but the morbidity of 65% is high. The most frequent morbidities (pneumopathy—23% and wound infection—20%) are of a similar percentage to other series [4]. Overall, 41% of patients had obstructive cancer with a risk of bronchopulmonary aspiration and 36% had peritonitis with implicit abdominal wall contamination and risk of postoperative wound infection; 20% of patients had diabetes with risk of wound infection; 10% had preoperative pulmonary problems that can lead to postoperative pneumonia. Although, it has not been found statistically that a long operating time can lead to a high rate of complications.

When to perform a Hartmann procedure or a primary anastomosis with or without a loop ileostomy is still controversial. There are multicenter studies that show no substantial difference between the two procedures. In most of the series in the literature, both for inflammatory diseases and for cancers, it is obvious that patients who presented with a high operative risk were operated using the Hartmann procedure, while the others underwent a sigmoid colectomy with or without ileostomy. The advantages of the Hartmann procedure are that it is a quick operation, and the risk of an anastomotic leak is practically avoided. Even if postoperative complications occur, they do not really compromise the general conditions of the patient and do not put the patient’s life in danger. The disadvantage of the Hartmann procedure is that the stoma is not reversed in many cases, especially in these critically ill patients. In general, the number of cases in the literature is small. Kreis et al. conducted an analysis of 789 patients that were treated. From the 73 patients who underwent emergency surgery, 36 received a primary anastomosis without stoma, 11 a primary anastomosis with loop ileostomy and 26 a Hartmann procedure. Mortality was 4% in the anastomosis group, 27% in the Hartmann procedure group and 12% overall, and recommended performing the Hartmann operation in severely ill patients who carried substantial comorbidity, considering the extent of peritonitis [16].

The mortality following primary anastomosis and anastomotic leakage performed in the emergency room remains much higher compared to the elective indication. However, colectomy with primary anastomosis is a more difficult procedure, which is not easily performed by all surgical teams and especially minimally invasive. The Hartmann operation was obviously reserved for the extremely ill patients [23,24,25,26,27].

The rate of 40% for the reversal of Hartmann procedure (Hartmann II) is lower than in most series [12,18]. In most statistics with Hartmann operations, diverticulitis cases predominate compared to colorectal cancers and present high-risk comorbidities, ASA III and IV, with comorbidities increasing and multiplying with age [28,29,30].

David et al. compared 3950 Hartmann procedures performed as an emergency and 1097 as an elective procedure. Only 23.3% of these patients underwent a reversal during the study period, with a median time interval between a Hartmann procedure and reversal of 284.5 days [17].

The reasons for the failure of the Hartmann II operation were recurrence, adhesion disease, short, atrophied rectal stump, sclerosis after radiotherapy, advanced age, associated conditions or the patient’s refusal.

Multiple trials have demonstrated the feasibility of laparoscopic Hartmann procedure, with comparable or even better results in terms of quality of life and disease-free interval and with reversal rates comparable to the open approach [31].

The laparoscopic approach to the Hartmann procedure is safe in elective surgery and in select emergent cases as well and shows a significantly lower overall complication rate with a shorter hospital stay, which is consistent with previous studies comparing laparoscopic and open approaches. Additionally, mortality is comparable between the two approaches [32,33,34].

Because there are insufficient data in the literature to support or refute the safety and efficacy of a laparoscopic approach vs. open, the minimally invasive approach should only be performed by an appropriately trained surgeon. The operative time is comparable or a little longer than open surgical resection, depending on the colon surgery experience of the surgical team, but laparoscopic surgery offers well-known advantages, especially in benign inflammatory conditions such as diverticulitis Hinchey III and IV [26,35,36].

Additionally, a Germany multicentric observation study compared different operative approaches in the emergency treatment of obstructive left-sided colon cancer, Hartmann procedure and primary anastomosis. Primary anastomosis in occlusive left colon carcinoma is indicated only in cases where the risk profile is favorable. In advanced occlusion and in high-risk cases, the Hartmann procedure should be used because the protective stoma appears to offer no advantage [37].

In recent years, self-expandable metal stents (SEMS) have been used more and more as a bridge to surgery for patients with obstructive colorectal cancer; they offer significant advantages, and they offer efficacy and safety comparable to HP in the management of malignant rectosigmoid obstruction, especially in people with high ASA risk, or in an advanced tumor stage, with faster recovery and shorter hospitalization time [38,39,40]. The use of SEMSs in the presence of perforation or peritonitis is contraindicated; in these conditions, a Hartmann resection is preferred [41].

## 5. Conclusions

The Hartmann procedure is still nowadays an operation widely used in emergency. We must not forget that the Hartmann Procedure can save lives even at the cost of a stoma reversal failure. Laparoscopy may become standard for the Hartmann procedure and the reversal of the Hartmann procedure, as was performed in the cases in this study, but the percentage of laparoscopy remains low due to advanced colorectal cancer, poor general condition both at the first and second intervention and the difficulties of the reversal of the Hartmann procedure.

## Figures and Tables

**Table 1:** Statistics for correlation between comorbidities and general complications.

Cardiac Comorbidities	General complications			95% Confidence Interval
	No	Yes	Total	M-L-Chi-square *χ*^2^
No	28	2	30	*χ*^2^ = 4.827 *p* = 0.023 Mark the differences Significant at *p* < 0.05
	93.3%	6.7%		
Yes	42	15	57	
	73.7%	26.3%		
Total	70	17	87	
**Pulmonary** **Comorbidities**	**General complications**			**95% Confidence Interval**
	No	Yes	Total	M-L-Chi-square *χ*^2^
No	63	4	67	*χ*^2^ = 6.014 *p* = 0.027 Mark the differences Significant at *p* < 0.05
	94%	10%		
Yes	15	5	20	
	75%	25%		
Total	78	9	87	

**Table 2:** Statistics for correlation between peritonitis and local complications.

Peritonitis	Local complications			95% Confidence Interval
	No	Yes	Total	M-L-Chi-square *χ*^2^
No	63	12	75	*χ*^2^ = 10.871 *p* = 0.003 Mark the differences Significant at *p* < 0.05
	84%	16%		
Yes	5	7	12	
	41.7%	58.3%		
Total	70	17	87	

**Table 3:** Statistical indicators of the number of excised nodes depending on the type of surgical approach.

Surgical Approach	Mean No Limphnodes	Media		Dev. Std.	Min	Max	Q25	Mediana	Q75
		*−95%*	*+95%*						
open	19.49	17.28	21.69	9.89	12	56			
laparoscopic	22.71	9.91	35.52	13.84	12	42			
Allgroup	19.75	17.57	21.92	10.20	12	56			

Levene Test of Homogeneity of Variances: F = 0.641; *p* = 0.426.

**Table 4:** Statistical indicators of the number of days of hospital stay depending on the type of surgical approach.

Surgical Abord	Mean Hospital Stays	Media		Dev. Std.	Min	Max	Q25	Mediana	Q75
		*−95%*	*+95%*						
open	12.24	10.60	13.88	7.37		38			
laparoscopic	5.29	2.91	7.66	2.56		8			
Allgroup	11.68	10.11	13.24	7.34		38			

Levene Test of Homogeneity of Variances: F = 6.107; *p* = 0.015.

**Table 5:** Hartmann procedure trend.

	Bradea (This Study)	Leong	Meyer	Kreis	David	Hallam
MEDIAN AGE (YEARS)	70	68	no	no	75	58
SEX RATIO B/F %	60/40	75/25	no	no	50/50	50/50
COMORBIDITIES %	70	79	no	100%	no	no
ASA III/II	73/27	38/31	no	26	no	84/36
COLORECTAL CANCER %	93	59	100	0	68.9	32
HOSPITAL STAY (DAYS)	12	13	no	no	19	8
MORTALITY %	21	19	7.5	27	20	7
REVERSAL OF HARTMANN PROCEDURE %	40	40	no	no	23.3	47

## Data Availability

The data published in this research are available on request from the first and last author and corresponding author.
